# Deficiency of *Vgll2* Gene Alters the Gene Expression Profiling of Skeletal Muscle Subjected to Mechanical Overload

**DOI:** 10.3389/fspor.2019.00041

**Published:** 2019-10-09

**Authors:** Keisuke Hitachi, Hidehito Inagaki, Hiroki Kurahashi, Hitoshi Okada, Kunihiro Tsuchida, Masahiko Honda

**Affiliations:** ^1^Division for Therapies against Intractable Diseases, Institute for Comprehensive Medical Science, Fujita Health University, Toyoake, Japan; ^2^Genome and Transcriptome Analysis Center, Fujita Health University, Toyoake, Japan; ^3^Department of Biochemistry, Faculty of Medicine, Kindai University, Osaka-Sayama, Japan; ^4^Department of Bioscience and Genetics, National Cerebral and Cardiovascular Center Research Institute, Suita, Japan

**Keywords:** skeletal muscle adaptation, resistance training, synergistic ablation, RNA sequencing, vestigial-like 2

## Introduction

Adult rodent skeletal muscles are composed of four types of myofibers (Schiaffino and Reggiani, 1996, 2011; Bassel-Duby and Olson, 2006), which are present in different proportions in different muscles. Slow-twitch (type I) fibers have fatigue resistance properties by virtue of large numbers of mitochondria. The remaining three types are fast-twitch (type II) fibers, subclassified as types IIA, IIX, and IIB. Of these, type IIA fibers exhibit the slowest shortening velocity and display fatigue resistance due to a high density of mitochondria. Conversely, type IIB fibers have the fastest shortening velocity and exhibit exercise intolerance due to a low density of mitochondria, while type IIX fibers are intermediate. Although the predominant fiber types in each muscle are determined during embryonic and fetal myogenesis (Lu et al., 1999; Agbulut et al., 2003), functional adaptations can lead to the alteration of these proportions through changes in gene expression (Swoap et al., 2000; Oh et al., 2005).

Vestigial is a nuclear protein that is essential for *Drosophila* wing formation and muscle differentiation (Paumard-Rigal et al., 1998; Simon et al., 2016). Four mammalian vestigial-like proteins (Vgll1, Vgll2, Vgll3, and Vgll4) have been identified, which support the function of TEA domain (TEAD) transcription factors in a tissue-specific manner (Maeda et al., 2002; Mielcarek et al., 2002; Pobbati and Hong, 2013). One of these proteins, Vgll2/VITO-1, is expressed in adult skeletal muscles (Maeda et al., 2002; Mielcarek et al., 2002) and plays a role in *in vitro* muscle differentiation (Maeda et al., 2002; Günther et al., 2004). Our previous study using *Vgll2* knockout (KO) mice revealed that Vgll2 regulates skeletal muscle fiber composition by repressing the neonatal expression of sex-determining region Y (SRY)-box 6 (Sox6), trans-acting transcription factor 3 (Sp3), and purine-rich element-binding protein B (Purβ), which are transcriptional repressors of slow-twitch fiber-related genes (Honda et al., 2017).

In humans, long-term resistance training not only induces muscle hypertrophy but also increases the proportion of slower muscle fibers by increasing and decreasing the number of type IIA and type IIX fibers, respectively (Hather et al., 1991; Adams et al., 1993; Williamson et al., 2001; Bickel et al., 2011). Like resistance training, mechanical overload (MOV, also called chronic overload) by synergistic ablation of the soleus and gastrocnemius muscles induces hypertrophy in the plantaris muscles. MOV also modifies the fiber type proportions in the plantaris muscles toward slower fibers by shifting type IIX and IIB fibers to type I and IIA fibers, through the activation of multiple genes related to muscle contractility in mice (Karasseva et al., 2003; Ji et al., 2007; McGee et al., 2008; Pérez-Schindler et al., 2013). We previously showed that MOV increases Vgll2 protein expression and activity, enhancing the functional adaptation of skeletal muscle (Honda et al., 2019). In addition, mice lacking *Vgll2* exhibited limited fiber type transition after MOV due to the repression of slow muscle genes, which are increased by MOV in wild-type mice. *Vgll2* KO mice also displayed downregulated expression of several genes involved in oxidative metabolism. Thus, Vgll2 is essential for the fast-to-slow shift of muscle fibers after MOV and acts by regulating the expression of downstream genes at the transcriptional level (Honda et al., 2019). However, gene expression changes downstream of Vgll2 in skeletal muscle subjected to MOV have not been systematically identified. To examine molecular network changes in skeletal muscle after MOV, we conducted a comparative gene expression analysis in wild-type and *Vgll2* KO mice under both sedentary and MOV conditions. Raw data files have been deposited to the Sequence Read Archive of the DNA Data Bank of Japan (DDBJ) under Accession No. DRA008472.

## Methods

### Animals

*Vgll2* KO mice and wild-type littermates on a C57BL/6J background were generated as previously described (Honda et al., 2017) and housed in cages at 24°C with a 12:12-h light–dark cycle. Animal experiments were approved by the Animal Care and Use Committee of the National Cerebral and Cardiovascular Center in Japan and conducted under institutional and national guidelines.

### Synergistic Ablation

MOV was performed as previously described (McGee et al., 2008; Honda et al., 2019). In brief, for synergistic ablation of the plantaris, the soleus and gastrocnemius were surgically removed from 10-weeks-old male wild-type littermates (Wild_MOV) and *Vgll2* KO mice (KO_MOV) under anesthesia (2.5% isoflurane). Mice that underwent sham surgeries were used as controls (Wild_sham, KO_sham). To explore the results of long-term effects of MOV, 6 weeks after surgery, the mice were sacrificed, and the plantaris muscles were collected and submerged in RNAlater (Thermo Fisher Scientific, Waltham, MA, USA) for RNA isolation.

### RNA Isolation and Library Preparation

Total RNA was extracted from the plantaris muscles using the miRNeasy Mini Kit (QIAGEN, Hilden, Germany) according to the manufacturer's instructions and quantified using a NanoDrop spectrophotometer (Thermo Fisher Scientific). Poly(A)+ RNA was purified from 1 μg of total RNA using the NEBNext Poly(A) mRNA Magnetic Isolation Module (New England Biolabs, Ipswich, MA, USA) and used for RNA sequencing (RNA-Seq) library preparation with the NEBNext Ultra RNA Library Prep Kit for Illumina (New England Biolabs), according to the manufacturer's protocol. The quantity of the libraries was assessed using a Library Quantification Kit (Takara, Shiga, Japan) and a Thermal Cycler Dice Real Time System TP800 (Takara); their quality was assessed with a DNA 1000 Kit and a Bioanalyzer 2100 (Agilent, Santa Clara, CA, USA).

### Transcriptome Analysis

RNA-Seq libraries were sequenced with 124-bp single-end reads using a HiSeq 1500 system (Illumina, San Diego, CA, USA) at Fujita Health University. Two biological replicates were performed per sample. Base calling was conducted using bcl2fastq ver. 1.8.4 software. The RNA-Seq raw data have been deposited in the DDBJ Sequence Read Archive under Accession No. DRA008472. DDBJ accession IDs for each sample are listed in Table 1. Raw sequence data were cleaned using FastQC ver. 0.11.3 software (https://www.bioinformatics.babraham.ac.uk/projects/fastqc/) and the command “–Q 33 –t 20 –l 30”. Trimmed reads were aligned to the mouse reference genome (mm10) using HISAT2 ver. 2.0.5 (Pertea et al., 2016) using default parameters. SAMtools ver. 1.3.1 software (Li et al., 2009) was used to convert SAM files, which contain aligned reads, into BAM files. A summary of sample names, raw reads, and aligned reads is provided in Table 1.

**Table 1:** Summary of the sequencing results.

**Sample name**	**Raw reads**	**Reads after trimming**	**Aligned reads**	**Overall alignment rate**	**DDBJ accession IDs**
Wild_MOV-1	26,019,105	25,982,454	25,189,725	96.95%	DRR180073
Wild_MOV-2	24,455,198	24,420,404	23,695,197	97.03%	DRR180074
Wild_sham-1	36,918,704	36,861,698	35,629,942	96.66%	DRR180075
Wild_sham-2	27,862,191	27,822,881	26,968,955	96.93%	DRR180076
KO_MOV-1	27,991,076	27,950,916	27,013,374	96.65%	DRR180077
KO_MOV-2	25,265,455	25,229,254	24,520,609	97.19%	DRR180078
KO_sham-1	28,007,495	27,968,083	27,040,208	96.68%	DRR180079
KO_sham-2	25,423,616	25,387,787	24,472,197	96.39%	DRR180080

### Statistical Analysis of Differentially Expressed Genes

Statistical analysis of RNA-Seq data was performed as previously described (Hitachi et al., 2019). In brief, to count the aligned reads, HTSeq ver. 0.6.0 software (Anders et al., 2015) was used against the Mus_musculus_UCSC_mm10.gtf file with the optional command “–stranded=no –format=bam”. We compared gene expression levels in Wild_sham mice with those in Wild_MOV, KO_sham, and KO_MOV mice using DESeq2 software ver. 1.12.4 (Love et al., 2014) using the Wald test. Differentially expressed genes with a false discovery rate (adjusted *P*-value, *p*_adj_) <0.001 and log2-fold change >1 or <-1 were considered significant. The expression levels of all genes in each experimental group are listed in Table S1.

**Figure 1:**
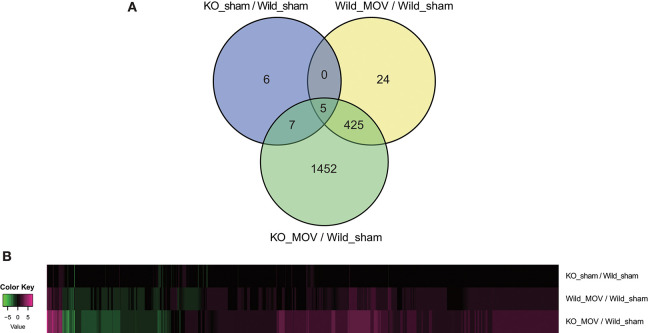
Summary of differentially expressed genes in the skeletal muscle of *Vgll2* knockout (KO) mice subjected to mechanical overload (MOV). **(A)** Venn diagram of differentially expressed genes between sets of experimental groups. **(B)** Heat map displaying differentially expressed genes in each experimental group (log2 ratio scale).

Using these parameters, we identified 454 differentially expressed genes (398 upregulated and 56 downregulated) after MOV in wild-type mice. Although in the absence of MOV, statistically significant expression changes were observed in only 18 genes (11 upregulated and 7 downregulated) in *Vgll2* KO mice compared to wild-type mice; in the presence of MOV, the expression levels of 1,889 genes (1,336 upregulated and 553 downregulated), including a majority of genes whose expression was altered by MOV in wild-type mice, were significantly altered in *Vgll2* KO mice compared with wild-type mice subjected to the sham operation (Figure 1A). Intriguingly, although Vgll2 is required to activate the expression of genes involved in the shift from fast to slow muscle fibers by MOV (Honda et al., 2019), Vgll2 deficiency eventually enhanced the effects of MOV on gene expression (Figure 1B). Therefore, these results suggest that Vgll2 plays a role in the functional adaptation of skeletal muscle by MOV beyond the fast-to-slow shifting of muscle fibers.

These data will be helpful in elucidating the skeletal muscle molecular networks that respond to MOV and allow in-depth analysis of expression changes in genes regulated by Vgll2 under both sedentary and MOV conditions. Interestingly, fusion genes involving *Vgll2* have been identified in the spindle cell variant of rhabdomyosarcoma (Alaggio et al., 2016). Thus, our data identifying genes downstream of *Vgll2* could also help reveal the molecular mechanisms underlying not only MOV but also human diseases caused by *Vgll2* fusion genes.

## Data Availability Statement

The RNA-Seq raw data have been deposited in the DDBJ Sequence Read Archive under Accession No. DRA008472 (https://ddbj.nig.ac.jp/DRASearch/study?acc=DRP005135).

## Ethics Statement

The animal study was reviewed and approved by the Animal Care and Use Committee of the National Cerebral and Cardiovascular Center in Japan.

## Author Contributions

KH, HI, HK, HO, and KT conducted the experiments. KH and MH designed the experiments and prepared the manuscript.

### Conflict of Interest

The authors declare that the research was conducted in the absence of any commercial or financial relationships that could be construed as a potential conflict of interest.

## Supplementary Material

Table S1 Plantaris RNA-Seq results.
